# Carotid intervention: stent or surgery? A prospective audit

**Published:** 2009-12

**Authors:** JV Robbs, T Mulaudzi, N Paruk, B Pillay, P Rajaruthnam

**Affiliations:** Vasular Unit, Department of Surgery, Faculty of Health Sciences, University of KwaZulu-Natal, Durban; Vasular Unit, Department of Surgery, Faculty of Health Sciences, University of KwaZulu-Natal, Durban; Vasular Unit, Department of Surgery, Faculty of Health Sciences, University of KwaZulu-Natal, Durban; Vasular Unit, Department of Surgery, Faculty of Health Sciences, University of KwaZulu-Natal, Durban; Vasular Unit, Department of Surgery, Faculty of Health Sciences, University of KwaZulu-Natal, Durban

## Abstract

**Summary:**

This study represents a prospective audit comparing carotid artery stenting (CAS) with carotid endarterectomy (CEA), performed by a single surgical team. Between January 2005 and December 2008, 440 patients were referred; 177 had CAS and 263 CEA. Selection of procedure was individualised and contra-indications for CAS included internal carotid artery (ICA) stenosis > 85–90%, intraluminal thrombus, ICA tortuosity, gross surface ulceration of plaque and excessive calcification. Type III aortic arch and arch calcification also precluded CAS.

Standard techniques were used for both procedures with a protection device routinely used for CAS. Most CEAs were performed under general anaesthesia, with selective intraluminal shunting. One hundred and eighty-six patients were selected for CAS; nine (48%) were converted to CEA for technical reasons.

The operative risk profile was similar, but significantly more in the CAS group were hypertensive. Almost half (49%) in the CAS group were asymptomatic vs 26% in the CEA group. All asymptomatics had 70+% stenosis on Duplex Doppler.

Results were reported within one month of the procedure. The stroke rate was 2.3% for CAS vs 1.9% for CEA (*p* > 0.05). Stroke and death plus one M1 was 4.5% after CAS vs 3.4% after CEA (*p* > 0.05). Disabling stroke occurred in 1.1% of CAS patients vs 0.4% of CEA patients. These results are satisfactory and compare favourably with other similar series.

## Summary

Carotid endarterectomy has long been established as the gold standard for the treatment of carotid bifurcation disease and possibly the best-studied procedure of all. The guidelines for the indications for surgery in symptomatic patients emerged in 1991 with the ECST and NASCET^1^,^2^ studies. Asymptomatic stenosis was addressed by the ACAS and ACST trials.^3^,^4^

Carotid artery stenting (CAS) is a relatively recent modality and the first randomised trial comparing carotid endarterectomy (CEA) with carotid artery stenting was abandoned after 17 patients had been treated, due to the unacceptable stroke rate in the stent group.^5^ Since then the technology and techniques have improved and randomised trials have emerged with acceptable short-term results.^6^,^7^

This study was not a randomised trial, but was an audit of the results of patients selected for either carotid artery stenting or carotid endarterectomy, both performed by the same surgical team. The results reported were within one month of surgery.

## Methods

The clinical and demographic data on all patients referred to the Vascular Unit at Inkosi Albert Luthuli Hospital, Durban for carotid intervention over a four-year period extending from January 2005 to December 2008 were prospectively recorded on a computerised database.

All patients were submitted to carotid Duplex Doppler studies. Computerised tomographic (CT) angiography was performed selectively on all patients with Duplex scans that were difficult to interpret or who had suspected aortic arch disease on clinical grounds, or to confirm suspected internal carotid artery occlusion reported on Duplex. The CT scan of the brain was performed on patients with a fixed neurological deficit, and all those with atypical neurological symptoms, according to our standard unit protocol.

All patients had full cardiac risk assessment, which included history, ECG and echocardiography. When indicated, stress ECG and coronary angiography were performed. Routine blood investigations included full blood count, plasma electrolyte and creatinine levels. Once referral had been made for the intervention, the vascular surgical team made the selection for either carotid artery stent or carotid endarterectomy at a weekly assessment meeting.

Contraindications for CAS in terms of the appearance of the plaque were internal carotid artery (ICA) stenosis of more than 85 to 90%, the presence of intraluminal thrombus, ICA tortuosity, gross surface ulceration of the plaque and excessive calcification. In terms of the aortic arch, excessive branch angulation (type III) and calcification were absolute contraindications. Relative contraindications were total occlusion of the opposite ICA, the presence of a bovine arch configuration (especially in relation to left ICA lesions, and advanced age (> 80 years). The latter was largely dependent on quality-of-life assessment.

## Carotid artery stent

Access was made via the groin and routine arch angiography was performed to confirm the configuration and suitability of the lesion. A protection device was routinely used. The patient was systemically heparinised and self-expanding Nitinol stents were used. In the majority of patients the Cordis® systems were used. For all patients, an anaesthetist was in attendance with arterial line monitoring of blood pressure. Postoperatively, the patient was returned to the intensive care unit (ICU) for a period of 24 hours.

## Carotid endarterectomy

In most patients, neurolept anaesthesia with loco regional infiltration was performed. Some surgeons preferred local anaesthetic with cervical plexus block. The operation followed a standard technique, most arteriotomies were closed without a patch and the patients were systemically heparinised. There is a policy of selective shunting using stump pressure measurement. Those with blood pressure less than 50 mmHg (mean) or with neurological symptoms developing while under local anaesthetic were shunted. All patients were routinely returned to the ICU. Results were reported within 30 days of the procedure and all statistical analysis was done using the chi-squared test.

## Results

Four hundred and forty patients were referred for carotid intervention;177 were successfully stented and 263 were treated by carotid endarterectomy.

## Carotid artery stents

One hundred and eighty-six patients were originally selected for CAS; nine (4.8%) were abandoned during the procedure. Two had a cardiac episode during angiography (angina). In two patients the plaque was eccentric, with marked surface ulceration and possible superimposed thrombus. Five patients had a bovine or type III arch configuration, which made selection and cannulation of the vessels hazardous. All of the above patients were eventually submitted for CEA. One hundred and seventy-seven patients were successfully stented.

Six patients (3.4%) developed severe intracerebral vasospasm, which responded to direct trinitryl infusion. Eight patients (4.5%) developed profound hypotension at the time of balloon dilatation of the stent, which responded to glycopyrrholate infusion. One of these patients developed an intra-procedural myocardial infarct (MI).

## Carotid endarterectomy

Two hundred and sixty-three patients underwent successful endarterectomy; 95 had the procedure performed under local anaesthetic. Sixty-six (25%) of these required an intraluminal shunt; 84 (32%) had a patch closure due to a small-calibre internal carotid artery (ICA). There were no significant intra-operative complications.

## Demographics

In the CAS group there were 94 males (53%), opposed to 186 males (71%) in the CEA group. The comparative age range is shown in Table 1. The majority of the patients were in the seventh decade (60–70 years) with a total of seven octogenerians. Race distribution can be seen in Table 2. This was similar in the cohorts, the majority being white or Asian. There were few Africans in either group.

**Table 1:** Age Range (Years)

	*CAS (n = 177)*		*CEA (n = 263)*	
*Range (years)*	*n*	%*	*n*	%*
40–50	0		21	8
50–60	44	25	47	18
60–70	80	45	145	55
70–80	51	29	45	17
80–90	2	1	5	2

*Percentages to nearest whole number.

**Table 2:** Racial Distribution

*Population group*	*CAS (n = 177)*		*CEA (n = 263)*	
	*n*	%*	*n*	%*
White	91	51	124	47
Asian	80	45	118	45
Coloured	3	2	12	5
African	3	2	9	3

*Percentages to nearest whole number.

## Operative risk profile

Table 3Table 3 shows the operative risk profile in both groups. There was a significant difference in the proportion of patients with hypertension, with 86% in the CAS group opposed to 58% in the CEA group (*p* < 0.001).

**Table 3:** Risk Profile

	*CAS (n = 177)*		*CEA (n = 263)*		
	*n*	%*	*n*	%*	
Smoking	61	34	119	45	*p* > 0.05
Hypertension	152	86	153	58	*p* < 0.001
Ischaemic heart disease	50	28	83	31	
Diabetes	71	40	90	34	
Hyperlipidaemia	22	12	28	11	
Chronic obstructive pulmonary disease	10	6	17	6	
Renal failure	6	3	3	1	

*Percentages to nearest whole number.

Although there was a higher proportion of smokers among the CEA patients, the difference was not statistically significant. Other major risk factors such as ischaemic heart disease, diabetes, hypolipidaemia, respiratory and renal disease showed no statistical difference.

## Clinical presentation

This is summarised in Table 4, where it can be seen that 49% of the CAS patients were asymptomatic, opposed to 26% of the CEA group. On Duplex Doppler assessment, all asymptomatic patients had carotid stenosis in excess of 70% using NASCET criteria. Among symptomatic patients, 80% or more had stenosis in excess of 70%. A small proportion in both groups had total occlusion of the opposite carotid artery (Table 5).

**Table 4:** Clinical Presentation

	*CAS (n = 177)*		*CEA (n = 263)*		
*Symptomatic*	*n*	%*	*n*	%*
TIA	54	29	113	43
Amaurosis	19	11	9	3
Stroke	18	10	72	27
Total	92	51	194	74
Asymptomatic	86	49	69	26

*Percentages to nearest whole.

**Table 5:** Percentage Stenosis On Duplex Doppler Evaluation

	*% Patients*	
*% Carotid stenosis*	*CAS (n = 177)*	*CEA (n = 263)*
Asymptomatic > 70	100	100
Symptomatic 50–70	20	15
70+	80	85
Total opposite occlusion	3	4

## Results within one month of procedure

Table 6 shows neurological and cardiac morbidity and all-cause mortality. There was no significant difference between the two treatment groups, with a stroke and stroke-plus-death rate of 2.3% in the CAS group and 1.9% after CEA (*p* > 0.05). The total stroke-plus-death-plus-myocardial infarction rate was marginally higher in the CAS group (4.5%), opposed to 3.4% in the CEA group (*p* > 0.05). Postoperative cerebral hyperperfusion syndrome occurred in a small proportion of patients after both procedures.

**Table 6:** Results Within One Month Of Procedure: Neurological And Cardiac Morbidity And All-Cause Mortality

	*CAS (n = 177)*		*CEA (n = 263)*		
	*n*	*%*	*n*	*%*	
Stroke	4	2.3	5	1.9	*p* > 0.05
Death	2	1.1	5	1.9	
Stroke + death	4	2.3	5	1.9	
MI*	4	2.3	4	1.5	
Stroke + death + MI	8	4.5	9	3.4	*p* > 0.05
Disabling stroke	2	1.1	1	0.4	
Cerebral hyperfusion syndrome	1	0.6	2	0.8	

*Myocardial infarction.

Local complications are summarised in Table 7. Three patients (1.7%) developed a significant groin haematoma after CAS, while seven (2.7%) required re-exploration for neck haematoma after CEA. Seven additional patients developed cranial nerve neuropraxias, four involving the mandibular branch of the facial nerve and three of the hypoglossal nerve, which resolved within the month.

**Table 7:** Results Within One Month Of Procedure: Local Complications

	*CAS (n = 177)*		*CEA (n = 263)*	
	*n*	*%*	*n*	*%*
Groin haematoma	3	1.7	–	
Neck haematoma	–		7	2.7
Cranial nerve neuropraxia	–		7	2.7

## Discussion

Carotid artery stenting has been in clinical use for about three decades and the technology continues to develop. There have been three randomised, controlled trials comparing CAS with CEA.^6^-^8^ This report does not represent a randomised trial and the selection of patients for CAS was individualised. All procedures were performed in a single centre by the same team.

The SAPPHIRE trial was published in 2006.^6^ This was a multicentre trial specifically addressing high-risk patients. This was defined as those having one of the following; ischaemic heart disease, severe pulmonary disease, contralateral carotid artery occlusion, laryngeal nerve palsy, previous neck surgery or irradiation, recurrent stenosis, and age in excess of 80 years. Seventy per cent of these patients had asymptomatic disease; 159 patients had CAS and 151 had CEA. The groups were well matched in terms of risk factors. All had a protection device inserted as a routine. At 30 days, the stroke, MI or death rate was 4.4% after CAS and 9.9% after CEA, which was not significantly different. When pure stroke morbidity was analysed, this was 3.6% after CAS and 3.1% after CEA.

The SPACE trial was published in 2006.^7^ This was a well-designed, prospective, multicentre trial comparing CEA and CAS. All patients were symptomatic with carotid stenosis of more than 70%, and the patients were well matched in terms of risk factors. There were 595 CEA and 605 CAS procedures performed. There were no specific contraindications to CAS and no routine protection device was inserted. There was a 2.3% crossover to CEA. The 30-day stroke-plus-death morbidity was 6.4% after CAS and 6.34% after CEA. The conclusion was that CAS was not an inferior procedure.

The EVA 3 S trial was also published in 2008 and was a multicentre, randomised study on symptomatic patients with carotid stenosis of more than 60%. Plaque morphology did not influence selection and the patients were randomly assigned. There were similar risk factors in both groups, which comprised 259 CEA and 260 CAS patients and there was a 5% crossover rate. No routine protection was performed and this constitutes a criticism, as the protocol was changed in mid-trial when it was realised that the results were inferior in the CAS patients and this was thought to be due to the lack of a protection device. The 30-day stroke and death rate was 3.9% after CEA and 9.6% after CAS, and the trial was stopped on the strength of this significant difference in favour of surgery.

In the present study there was a reasonable match in terms of risk factors, with the exception of hypertension, which had a significantly higher prevalence in the CAS group. It was also of interest to note that the stent group had a higher proportion of asymptomatic patients, although this was not a specific criterion in the selection process. The stroke, and stroke-plus-death rate of 2.3% after CAS and 1.9% after CEA compares very favourably with all the above trials, as does the stroke death and MI rate of 4.5% and 3.4% after CAS and CEA, respectively, which compared well with the SAPPHIRE results.

It is of interest to note the relative rarity of significant carotid artery disease in the African population, which corroborates the findings of an earlier study on hospital prevalence of peripheral vascular disease in Durban.^9^

The general conclusion reached in the SAPPHIRE and SPACE trials was that CAS was not inferior to CEA. EVA 3 S showed a distinct advantage for carotid endarterectomy. CEA has been extensively studied and remains the gold standard for the treatment of carotid stenosis. The major advantage for CAS is the lack of surgical trauma, shorter hospitalisation time and general patient friendliness of the procedure, with a low complication rate, particularly cranial nerve palsies.

The dilemma that now arises is to determine which patients are not suitable for carotid artery stent insertion. The major cause of intra-operative neurological deficit is embolisation. The vulnerable periods for causing embolisation during the CAS procedure appear to be during selection of the arch branches, traversing the lesion, insertion of the protective device, passage of the stent, and during balloon dilatation. All phases have been shown to cause embolisation in *ex vivo* plaque models.^10^-^12^ An additional factor is the haemodynamic changes which may occur during balloon dilatation of the stent.

Common sense dictates that excessive angulation of the origins of the arch branches and tortuosity of the vessels create difficulties with selection and manipulation of guide wires, with the potential for dislodging particles. In addition, the internal carotid artery tortuosity may result in kinking at the distal end of the stent, with the potential for longer-term problems. It also seems obvious that morphology of the plaque would have some influence on intraprocedural embolisation, and histological studies have correlated symptomatic plaques with a soft lipid or haemorrhagic core and surface ulceration.^13^

Clinical evidence for plaques at risk of embolic complications during CAS is sparse. In a study using Duplex Doppler findings, Biasi *et al.*^14^ reported that there was a significantly higher neurological event rate after CAS in the presence of plaques with a soft lipid core associated with haemorrhage, as calculated by the grey-scale median method (GSM), with a value of less than 25 defined as the cut off.

Angiography is less reliable in defining plaque morphology but long and multiple stenoses seem to be predictors of problems during CAS.^15^,^16^ In the same study, angiographic evidence of the degree of stenosis surface ulceration or irregularity of the surface did not contribute to neurological complications. In general, there are no reliable guidelines for contraindications for stenting in terms of plaque morphology besides the presence of echolucency on Duplex Doppler scanning.

In our own practice Duplex Doppler has been the main tool for plaque selection, and severe stenosis of more than 85 to 90%, echolucency, surface ulceration or the presence of thrombus has precluded selection for CAS. Relative contraindications have been severe calcification, which may preclude dilatation, or eccentric plaque in which manipulation of the wire into the distal internal carotid might be difficult. In those considered for stenting the final decision was made at diagnostic arch angiography.

The presence of a type III configuration, excessive calcification or the presence of a bovine arch anomaly, particularly when the left carotid bifurcation was the target lesion, led to abandonment of the procedure. In the presence of a bovine arch, the right carotid is still quite easily accessible. In addition we have a low threshold for conversion to CEA if difficulties are encountered during wire manipulation. The endarterectomy is then performed by the same team. Conversion to CEA occurred in 4.8% of our patients. It is also in our practice to have an anesthetist present at the time of the procedure so that any cardiac or haemodynamic changes can be promptly detected and treated.

## Conclusion

CAS is now an established treatment modality for carotid bifurcation disease. The results in the above series are satisfactory but continued clinical audit and appraisal are essential in order to improve results.
